# Neighborhood Diversity Promotes Tree Growth in a Secondary Forest: The Interplay of Intraspecific Competition, Interspecific Competition, and Spatial Scale

**DOI:** 10.3390/plants13141994

**Published:** 2024-07-21

**Authors:** Haonan Zhang, Yuanyun Gao, Xiao Zheng, Yaping Hu, Xu Zhou, Yanming Fang, Yao Li, Lei Xie, Hui Ding

**Affiliations:** 1 Innovative Research Team for Forest Restoration Mechanisms, Chishui National Ecological Quality Comprehensive Monitoring Stations, Nanjing Institute of Environmental Sciences, Ministry of Ecology and Environment (MEE), Nanjing 210042, China; 2 Research Center for Nature Conservation and Biodiversity, State Environmental Protection Scientific Observation and Research Station for Ecology and Environment of Wuyi Mountains, State Environmental Protection Key Laboratory on Biosafety, Nanjing Institute of Environmental Sciences, Ministry of Ecology and Environment (MEE), Nanjing 210042, China; 3 Co-Innovation Center for Sustainable Forestry in Southern China, College of Life Sciences, Key Laboratory of State Forestry and Grassland Administration on Subtropical Forest Biodiversity Conservation, Nanjing Forestry University, Nanjing 210037, China

**Keywords:** tree growth, species diversity, intraspecific competition, interspecific competition, scale-dependent effects, secondary forests

## Abstract

Understanding the biodiversity–productivity relationship (BPR) is crucial for biodiversity conservation and ecosystem management. While it is known that diversity enhances forest productivity, the underlying mechanisms at the local neighborhood level remain poorly understood. We established a 9.6 ha dynamic forest plot to study how neighborhood diversity, intraspecific competition, and interspecific competition influence tree growth across spatial scales using linear mixed-effects models. Our analysis reveals a significant positive correlation between neighborhood species richness (NSR) and relative growth rate (RGR). Notably, intraspecific competition, measured by conspecific neighborhood density and resource competition, negatively impacts RGR at finer scales, indicating intense competition among conspecifics for limited resources. In contrast, interspecific competition, measured by heterospecific density and resource competition, has a negligible impact on RGR. The relative importance of diversity and intra/interspecific competition in influencing tree growth varies with scale. At fine scales, intraspecific competition dominates negatively, while at larger scales, the positive effect of NSR on RGR increases, contributing to a positive BPR. These findings highlight the intricate interplay between local interactions and spatial scale in modulating tree growth, emphasizing the importance of considering biotic interactions and spatial variability in studying BPR.

## 1. Introduction

The interplay between species diversity and ecosystem productivity, known as the biodiversity–productivity relationship (BPR), represents a cornerstone of ecological research. Understanding this relationship is crucial for comprehending the consequences of biodiversity loss on ecosystem functionality and services [1,2,3,4]. The insights gained from studying the BPR have profound implications for ecosystem function, conservation biology, and the sustainable management of natural resources in the face of global biodiversity decline [4,5,6,7].

Central to the BPR is the premise that increased species diversity leads to enhanced ecosystem productivity, a concept supported by extensive research across various ecosystems [1,5]. Empirical evidence supporting a positive BPR spans diverse landscapes, including grasslands, forests, and aquatic environments [6]. For instance, investigations in grassland ecosystems have shown that plots with increased species richness exhibit higher biomass production, directly indicating enhanced ecosystem productivity [2,4,6,7,8,9]. Similarly, forest ecosystems have demonstrated positive correlations between tree species diversity and productivity, with a notable relationship between biodiversity and tree growth performance [2,4,7,8,9,10]. The positive impact of biodiversity on tree growth is considered a significant characteristic of the biodiversity effect, widely observed not only in large-scale studies [2] but also at finer scales, such as at the neighborhood level where individual trees interact with their diversely constituted neighboring trees [7,8,9,10]. These findings across scales underscore the robustness of the biodiversity–productivity relationship, highlighting the critical role of species diversity in fostering ecosystem productivity not only at the community level but also in influencing individual organism interactions within their immediate environments at a fine spatial scale [7,8,9,10].

Although the relationship between species diversity and ecosystem productivity is widely supported, it exhibits considerable variability across different spatial scales. At larger scales, such as landscapes or biomes, the BPR tends to show more consistent patterns, with higher biodiversity generally associated with increased productivity. At these broad scales, the impact of species diversity on productivity is influenced by factors such as species turnover (beta diversity) and the distribution of functional traits across the landscape, which promote more efficient resource use and enhance ecosystem stability [11,12,13]. However, the variability introduced by local density-dependent interactions at finer scales complicates the generalizability of the observed positive correlations at broader scales. Studies suggest that the strength and even direction of this relationship can vary significantly with spatial scale, with finer scales showing more variability [1,13]. This scale-dependent variability is attributed to localized resource competition and the specific ecological niches occupied by different species, which may not be as apparent in large-scale studies [7,8,9,14]. Given these insights, it is imperative to further investigate the mechanisms through which local intra- and interspecific competition affect the biodiversity–productivity relationship, particularly at the neighborhood scale [9,10,15,16,17]. Therefore, developing multiscale models that accurately capture the complex interplay of diversity’s effects on ecosystem functioning across various scales is not only essential for advancing ecological theory and modeling but also crucial for understanding the more intricate neighborhood effects on the BPR [10,18,19,20,21,22].

At smaller scales, the variability in the species diversity–productivity relationship is significantly linked to neighbor effects, where both conspecific and heterospecific density dependence and resource competition may play a substantial role in modulating this relationship [7,8,9,15,16,17]. In forest communities, resources such as light and water are often limited, necessitating inevitable competition among individual trees with their neighboring conspecifics or heterospecifics as they grow [9,10,11]. The intensity of this competition is dependent on the density and size of adjacent trees [10]. On one hand, at the local scale, particularly at the fine scale of tree-to-tree interactions, neighboring tree individuals frequently experience intense intraspecific and interspecific competition [9,10,11,15,16,17], which is known as conspecific density dependence and has been widely documented in both tropical and subtropical forest communities [15,16,17]. On the other hand, the ability of plants of different sizes to acquire resources varies significantly. Larger trees are often more capable of capturing sunlight and accessing soil nutrients, thereby gaining a competitive advantage in resource acquisition [7,8,9,10,11]. Consequently, tree density and size markedly affect individual tree growth performance. However, the regulatory mechanisms and the relative importance of these effects on the species diversity–productivity relationship remain understudied. Therefore, incorporating the competitive effects of neighboring individuals into studies of the biodiversity–growth relationship is crucial, particularly at the neighborhood scale where these interactions are most pronounced.

**Figure 1:**
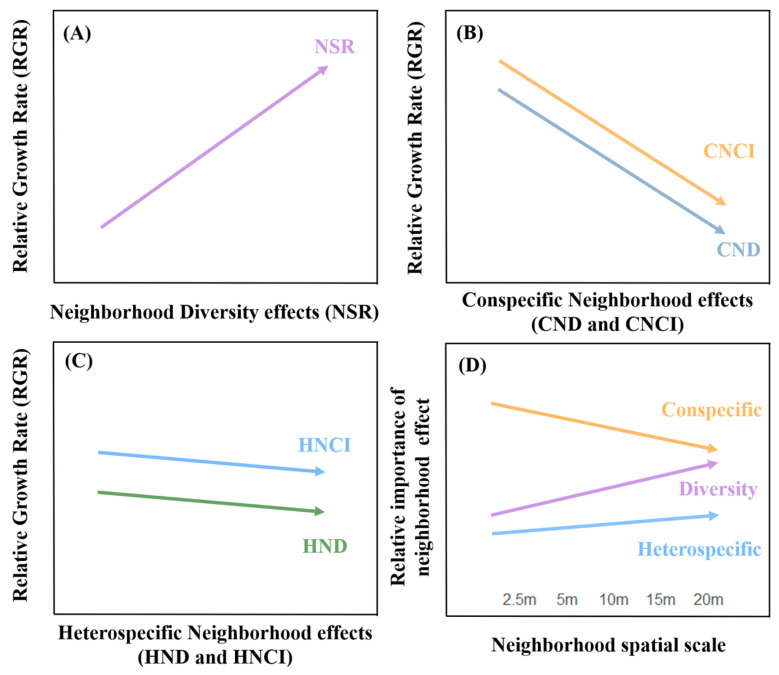
The research framework and scientific hypotheses. (i) In panel (**A**), Hypothesis 1 (H1) addresses the critical relationship between biodiversity effect and productivity. Specifically, we hypothesize a significant positive correlation between neighborhood species richness (NSR) and relative growth rate (RGR). (ii) In panels (**B**,**C**), Hypothesis 2 (H2) focuses on the roles of intraspecific and interspecific competition in shaping tree growth patterns in secondary forests. This hypothesis quantifies conspecific and heterospecific neighborhood density (CND and HND), as well as conspecific and heterospecific resource competition indices (CNCI and HNCI) at the neighborhood scale. We hypothesize that conspecific neighborhood effects (CND and CNCI) exert a pronounced negative impact on RGR at finer scales, whereas heterospecific neighborhood effects (HND and HNCI) are generally insignificant. (iii) In panel (**D**), Hypothesis 3 (H3) examines the relative importance of neighborhood diversity effects, conspecific, and heterospecific neighborhood effects. Specifically, we hypothesize that neighborhood diversity, density, and resource competition collectively regulate the relationship between species diversity and tree growth. The relative importance of these neighborhood effects is expected to vary across different spatial scales. At the tree-to-tree neighborhood scale, conspecific negative density dependence may dominate, while the biodiversity effect is anticipated to become increasingly important and exhibit a more pronounced positive effect on individual tree growth as the spatial scale increases.

In this context, our study quantified neighborhood species richness (NSR), conspecific and heterospecific neighborhood density (CND and HND), and conspecific and heterospecific resource competition indices (CNCI and HNCI) at the neighborhood scale (across five scale gradients from 2.5 m to 20 m). We linked these influencing factors with the individual trees’ relative growth rate (RGR) to estimate the effect sizes of different factors on tree growth at specific scales. This approach enabled us to quantitatively analyze how species diversity and intra- and interspecific competition collectively regulate individual tree growth. To validate our hypotheses, we established a 9.6-hectare dynamic monitoring plot in a secondary forest that had undergone severe anthropogenic disturbance 20 years prior. We conducted a comprehensive survey and repeated measurements every five years. Our hypotheses are as follows (Figure 1): Hypothesis 1 asserts that there is a significant positive correlation between neighborhood scale biodiversity and relative growth rate (RGR) during the early stages of secondary succession, demonstrating a beneficial biodiversity–production relationship. Hypothesis 2 proposes that in forests undergoing early secondary recovery following disturbance, conspecific negative density dependence (CNDD) is prevalent, likely exerting a negative impact on individual RGR. Furthermore, the influence of heterospecific negative density dependence is proposed to be less pronounced than that of intraspecific competition. Hypothesis 3 suggests that neighborhood diversity, density, and resource competition effects collectively regulate the relationship between species diversity and tree growth, with the relative importance of various neighborhood effects varying across different testing scales. At the tree-to-tree neighborhood scale, conspecific negative density dependence may dominate, while the diversity effect tends to become relatively more important and exhibits a more pronounced positive effect on individual tree growth as spatial scale increases. Exploring the local density-dependent interactions at the neighborhood scale and their impact on species diversity and ecosystem productivity is crucial for ecological theory and ecosystem management practices.

## 2. Results

### 2.1. Neighborhood Diversity Effects

**Figure 2:**
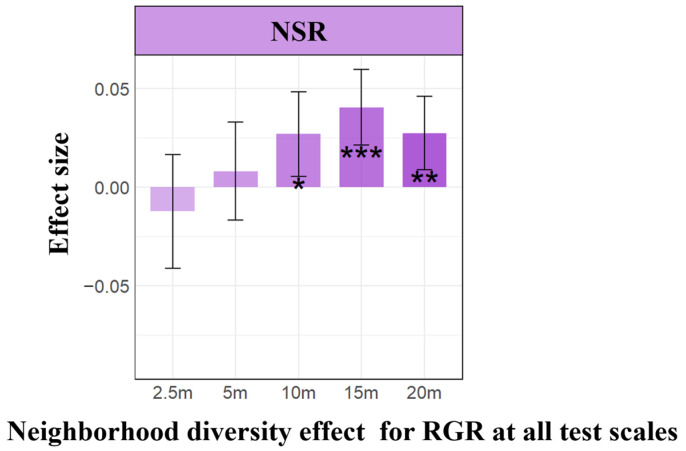
Parameter estimates of species diversity effects on relative growth rate (RGR) at neighborhood scales. The purple bar graphs depict the parameter estimation of neighborhood diversity richness (NSR) on the RGR of individual focal trees across different spatial scales. Positive values denote positive effects, while negative values signify negative effects. Significance levels are denoted by an asterisk (* *p* < 0.05; ** *p* < 0.01; *** *p* < 0.001).

**Figure 3:**
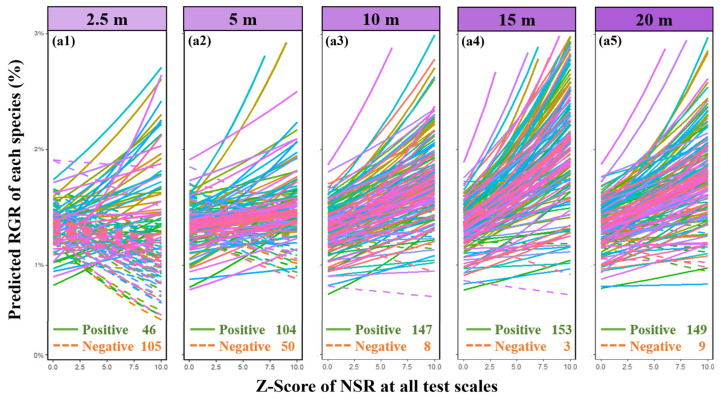
Multiscale relationship between species richness and relative growth rate (RGR) among all species. It displays annual diversity–RGR relationships for each of the 158 observed species at various spatial scales: 2.5 m (**a1**), 5 m (**a2**), 10 m (**a3**), 15 m (**a4**), 20 m (**a5**). Different species are represented by different colors in the lines (see Figure S1 in the supporting information), the solid line represents a positive correlation, and the dotted line represents a negative correlation. Predicted RGRs are back-transformed from the linear mixed model as described in the text, and all biodiversity effects were Z-score transformed at quantification. To enhance comparability and uniformity of the presentation results, we converted the Z-score values to positive in the figures; the untransformed original values can be found in Supplementary Figure S2.

Consistent with our initial hypothesis (Figure 1, H1), parameter estimates from linear mixed-effects models (LMMs) demonstrated a significant positive correlation between individual tree neighborhood diversity and their relative growth rate (Figure 2). Specifically, an increase in neighborhood diversity correlates with improved growth performance of individual trees, illustrating a positive biodiversity–productivity relationship at the scale of 5–20 m (positive significant relationship at the scale of 10–20 m). We observed a scale-dependent effect across different species (Figure 3), where the biodiversity–productivity relationship exhibited greater variability at smaller scales but gradually stabilized into a consistent positive correlation as spatial scale increased.

### 2.2. Intraspecific and Interspecific Competition

**Figure 4:**
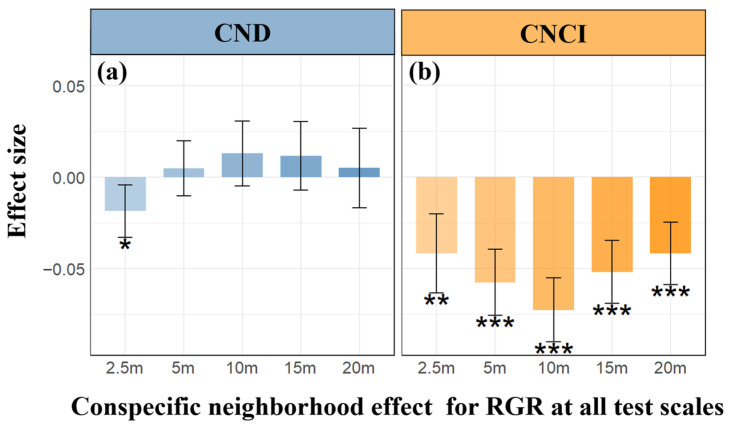
Parameter estimates for the effects of conspecific density and resource competition on relative growth rate (RGR) at neighborhood scales. The dark blue and orange bar graphs depict the parameter estimation of conspecific neighborhood density (CND) (**a**) and conspecific neighbor competition Index (CNCI) (**b**) on the RGR of individual focal trees across different spatial scales, respectively. Positive values denote positive effects, while negative values signify negative effects. Significance levels are denoted by an asterisk (* *p* < 0.05; ** *p* < 0.01; *** *p* < 0.001).

**Figure 5:**
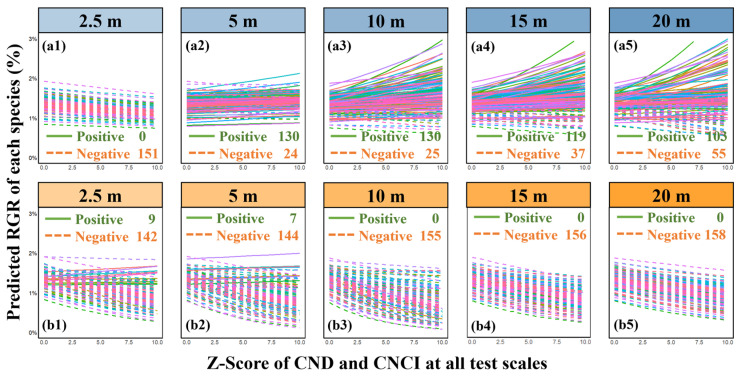
Multiscale relationship between conspecific density, resource competition, and relative growth rate (RGR) among all species. It displays annual conspecific neighborhood effect–RGR relationships for each of the 158 observed species at various spatial scales: 2.5 m (**a1**,**b1**), 5 m (**a2**,**b2**), 10 m (**a3**,**b3**), 15 m (**a4**,**b4**), 20 m (**a5**,**b5**). Different species are represented by different colors in the lines, the solid line represents a positive correlation, and the dotted line represents a negative correlation. Predicted RGR are back-transformed from the linear mixed model as described in the text, and all conspecific neighborhood effects were Z-score transformed at quantification. To enhance comparability and uniformity of the presentation results, we converted the Z-score values to positive in the figures; the untransformed original values can be found in Supplementary Figure S2.

**Figure 6:**
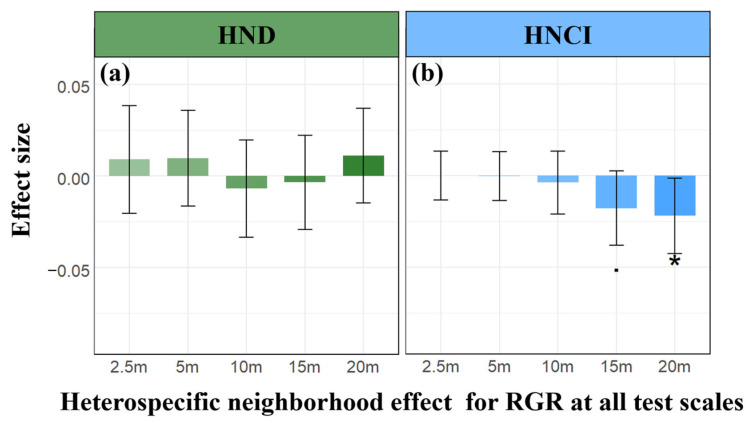
Parameter estimates for the effects of heterospecific density and resource competition on RGR at neighborhood scales. The green and light blue bar graphs depict the parameter estimation of heterospecific neighborhood density (HND) (**a**) and heterospecific neighborhood competition index (HNCI) (**b**) on the RGR of individual focal trees across different spatial scales, respectively. Positive values denote positive effects, while negative values signify negative effects. Significance levels are denoted by an asterisk (*) or a dot (·) (· *p* < 0.05; * *p* < 0.1).

**Figure 7:**
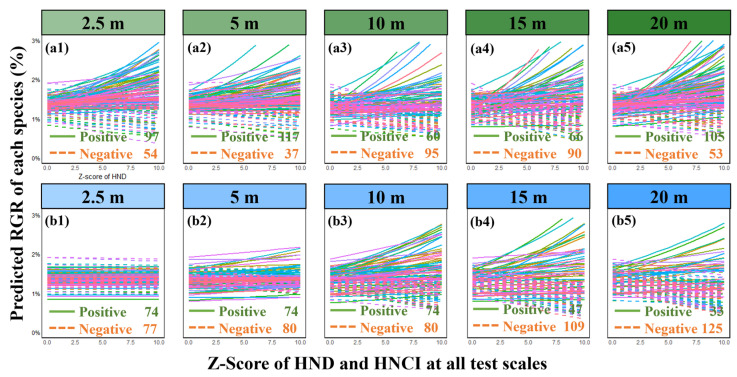
Multiscale relationship between heterospecific density, resource competition, and relative growth rate (RGR) among all species. It displays annual heterospecific neighborhood effect–RGR relationships for each of the 158 observed species at various spatial scales: 2.5 m (**a1**,**b1**), 5 m (**a2**,**b2**), 10 m (**a3**,**b3**), 15 m (**a4**,**b4**), 20 m (**a5**,**b5**). Different species are represented by different colors in the lines, the solid line represents a positive correlation, and the dotted line represents a negative correlation. Predicted RGR values are back-transformed from the linear mixed model as described in the text, and all heterospecific neighborhood effects were Z-score transformed at quantification. To enhance the comparability and uniformity of the presentation results, we converted the Z-score values to positive in the figures; the untransformed original values can be found in Supplementary Figure S2.

Results from linear mixed-effects models (LMMs) reveal a significant and scale-varying quantitative relationship between intraspecific neighborhood effects and RGR. Specifically, at a small scale (2.5 m), both conspecific neighbor density (CND) and conspecific neighbor competition index (CNCI) consistently exhibited a significant negative impact on RGR (Figure 4a,b). Across scales from 5 m to 20 m, CND showed a positive but not significant correlation with RGR, with substantial variability among different species (Figure 5(a1–a5)). CNCI maintained a significant negative correlation with RGR across all scales (Figure 5(b1–b5)), displaying a uniform trend among 158 species within a 20 × 20 m scale. However, the detection of heterospecific neighborhood effects (HND and HNCI) was largely insignificant. Despite observing a significant negative correlation between HNCI and RGR at scales of 15 m and 20 m (Figure 6b), many species demonstrated specificity in their responses (Figure 7(b1–b5)). The relationship between HND and RGR was not significant across all scales (Figure 6a), with no discernible trend and considerable variability among different species (Figure 7(a1–a5)). As we mentioned in Hypothesis 2 (H2), it can be inferred that the intensity of interspecific competition in this study is significantly lower than that of intraspecific competition, and it may represent one of the mechanisms by which diversity in secondary forests rapidly recovers during early successional stages.

### 2.3. Relative Importance of Diversity and Density Effect across Fine Spatial Scales

**Figure 8:**
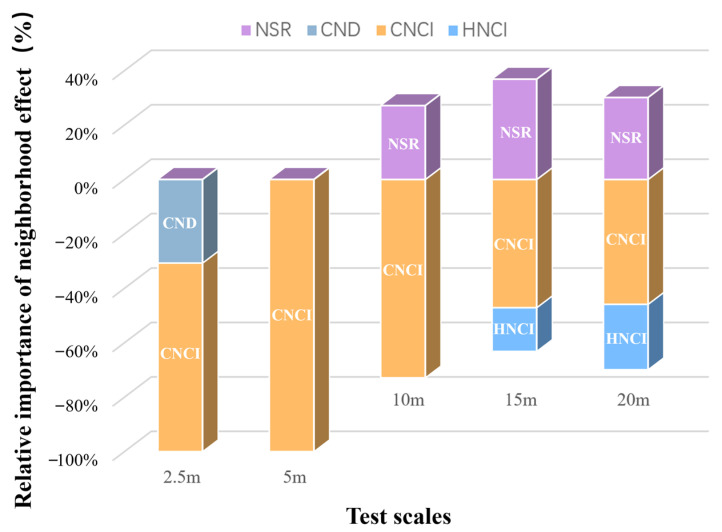
Relative importance of neighborhood effects at all test scales. This figure displays the relative effect sizes of neighborhood species richness (NSR), conspecific negative density (CND), conspecific neighborhood competition index (CNCI), and heterospecific neighborhood competition index (HNCI) on the relative growth rate. The graph only includes significant effects; since heterospecific negative density (HND) was not significant at any test scale in this study, it is not included in the calculations of relative importance. The relative effect of each predictor (neighborhood effect) and their interactions is calculated as the ratio of its parameter estimate to the sum of all parameter estimates.

As we expected in Hypothesis 3 (H3), neighborhood effects such as neighborhood species richness (NSR), conspecific negative density dependence (CNDD), neighborhood competition index (NCI), and heterospecific neighborhood competition index (HNCI) collectively regulate the relationship between species diversity and productivity. Among these, only NSR exhibits a positive effect on the growth of focal tree species (Figure 8).

However, the relative importance of various neighborhood effects varies with different testing scales. At a neighborhood scale of 5 m, we observed that neighborhood effects were overwhelmingly dominated by negative effects due to conspecific density or resource competition, making it difficult to discern a significant positive diversity–productivity relationship. However, as spatial scale increased, the relative importance of neighborhood species richness (NSR) became more pronounced at scales of 10 to 20 m, accounting for approximately 20% to 40% of the total effect and contributing to a positive diversity–productivity relationship. Additionally, at scales exceeding 15 m, interspecific resource competition led to a reduction in growth, representing 15% to 20% of the total effect, though conspecific negative density dependence remained predominant. In summary, the negative impact of conspecific density and resource competition on individual tree growth was predominant. However, as the test scale increased, the detectability of interspecific neighborhood effects became more significant, particularly the NSR effect at scales of 10 to 20 m, which facilitated a positive diversity–productivity relationship.

## 3. Discussion

The intricate relationship between biodiversity and ecosystem productivity, as highlighted by our research within the context of secondary forests, underscores a pivotal consensus: species diversity enhances ecosystem productivity. This understanding, deeply rooted in the foundational studies by Tilman et al. (2014) and Cardinale et al. (2012), is extended by our findings, which emphasize the crucial role of neighborhood-scale diversity [1,5]. Similar to earlier research at broader scales, such as landscapes or biomes, the biodiversity–productivity relationship (BPR) tends to display more consistent patterns, with higher biodiversity generally linked to increased productivity [11,12,23,24]. Our study also observed a similar phenomenon, namely an increase in tree growth rates with higher neighborhood diversity, within a neighborhood scale of 10–20 m (Figure 2), thus affirming a positive correlation between biodiversity and productivity. This observation provides tangible evidence of biodiversity in boosting ecosystem productivity at the neighborhood scale.

However, at smaller neighborhood scales (below 2.5 m), our research observes that the consistently positive diversity–productivity relationship noted at 10–20 m scales becomes more complex (Figure 3) and even exhibits a negative diversity–growth relationship at a scale of 2.5 m (Figure 2), although it is not statistically significant. We attribute this high variability and the non-significant negative relationship between plant diversity and growth at these smaller scales to the intricate interactions between the focal tree species and their neighboring individuals, particularly concerning conspecific density dependence and resource competition [7,8,9,10,15,16,17]. Additionally, the inclusion of random slopes for species enabled us to capture species-specific response variability, which is crucial for understanding the nuanced interactions at smaller scales. Our analysis of conspecific neighborhood effects (CND and CNCI) supports this view, particularly at very small scales below 2.5 m, where we found that neighborhood effects were overwhelmingly dominated by negative effects due to intraspecific competition (Figure 8), as indicated by the conspecific neighbor density (CND) and conspecific neighbor competition index (CNCI), which had a significant negative impact on RGR across various scales (Figure 4). This suggests that individuals of the same species exert a stronger competitive pressure on each other, likely due to direct competition for identical resources (light, water, and nutrients) and space [7,8,10,15,16,17,25,26]. Especially at very small scales, the limited space leads to the dominance of negative density effects among neighboring tree individuals (Figure 8), making it challenging to detect a positive diversity–growth relationship. On the other hand, interspecific competition, as measured by the heterospecific neighbor density (HND) and heterospecific neighbor competition index (HNCI), showed a less significant impact on RGR (Figure 6). This finding highlights the importance of niche differentiation in mediating competition among coexisting species [7,9,10,17]. According to niche theory, species coexistence is facilitated by differences in resource use and habitat preferences, which reduce direct competition and allow for a more equitable distribution of resources among species [27,28,29,30].

Interestingly, as spatial scale increases, the relative importance of neighborhood species richness (NSR) at scales of 10 m to 20 m increases, accounting for up to approximately 40% of the total neighborhood effect, thereby contributing to a positive biodiversity–productivity relationship. These results underscore the complexity of ecological interactions and the role of spatial context in mediating these interactions, indicating that at smaller scales, intense competition for resources may overshadow the positive effects of species diversity on productivity [7,8,9,10,17,31]. In contrast, at broader scales, the benefits of species diversity, possibly through mechanisms such as niche complementarity and reduced competition, become more apparent. Our research further elucidates the scale-dependent dynamics of the biodiversity–productivity relationship, a topic increasingly emphasized in recent ecological studies. Huang et al. (2018) and Liang et al. (2016) demonstrated that the impacts of biodiversity on ecosystem functioning can vary significantly across large spatial scales [4,32]. This variability implies that the mechanisms through which biodiversity influences ecosystem productivity—such as niche differentiation and resource partitioning—may manifest differently depending on the spatial scale under investigation [7,9,10,33]. Our findings unequivocally support this perspective, demonstrating that the positive effects of biodiversity are not consistent across different tree species but rather are modulated by the spatial scale at which they are examined.

Additionally, we observed a scale-dependent effect among different species, whereby at smaller scales, the biodiversity–productivity relationship exhibits greater interspecific variation (Figure 3a1), but as spatial scale increases, this relationship gradually stabilizes into a consistent positive correlation (Figure 3a5). This suggests that different species may respond differently to neighborhood effects—although neighborhood tree species richness generally promotes individual tree productivity, species with different resource utilization and competition strategies may exhibit varying responses [7,8]. Specifically, resource-acquisitive species are often more susceptible to reductions in individual growth due to neighborhood competition at smaller spatial scales, while species with more conservative resource utilization strategies may benefit more from diversity effects in individual growth [7,9,10].

In summary, our research extends the scope of previous studies on the plant species diversity–productivity relationship by demonstrating that at very small scales, the combined effects of conspecific and heterospecific density dependence and resource competition can alter the previously stable positive biodiversity–productivity relationship observed at larger scales. Specifically, at tree-to-tree neighborhood distances of less than 2.5 m, tree growth is predominantly influenced by conspecific neighborhood effects, which adversely affect growth. Traditional views have emphasized the role of interspecific competition in driving community assembly and species distribution patterns [28]. However, our findings, along with recent studies [29,30], suggest that intraspecific competition may play an equally, if not more, significant role in influencing plant community dynamics and ecosystem functioning. Understanding the differential impacts of intraspecific and interspecific competition on tree growth and the biodiversity–productivity relationship is crucial for gaining insights into the dynamics of secondary forest ecosystems. Moreover, the dominance of intraspecific competition, particularly in the early stages of forest succession, may significantly influence patterns of species recruitment, growth, and mortality. This, in turn, shapes the trajectory of forest development and recovery [9,10,11,31,32]. Therefore, recognizing the importance of intraspecific competition provides a more comprehensive understanding of forest ecology and the factors driving ecosystem resilience and productivity. Our research also confirms that the positive effects of biodiversity are modulated by spatial scale and interspecific variability, emphasizing the importance of scale in understanding ecological phenomena and suggesting that processes observed at one scale may not be directly extrapolated to another [14,34]. This is critical for understanding biodiversity’s effects and underscores the importance of considering spatial scale in ecological research and ecosystem management practices. Recognizing the importance of intraspecific competition and the scale-dependent nature of biodiversity effects provides a more comprehensive understanding of forest ecology and the factors driving ecosystem resilience and productivity.

## 4. Materials and Methods

### 4.1. Data Collection

The research area is situated in the Wuyishan National Park, located in the northwestern part of Fujian Province, China. This region experiences an average annual temperature of 19.2 °C and receives about 1600 mm of rainfall yearly (Figure S3 in the Supplementary Files). It enjoys an average annual sunshine of 1910.2 h, with a frost-free season lasting between 227 and 246 days. The dominant natural vegetation in this locale is the subtropical evergreen broad-leaved forest [35], although extensive commercial logging has historically transformed many primary forests into secondary forests [9,36].

For our research, we established a 9.6-hectare (400 m × 240 m) dynamic observation plot (27°35′24.23″ N, 117°45′55.43″ E) within the subtropical evergreen broad-leaved secondary forest, covering dimensions of 400 m by 240 m (Figure S4 in the Supplementary Material). This plot lies at an altitude that varies from 450 to 580 m, exhibiting minimal topographical variation. The long axis of the plot runs parallel to the main ridge in a northeast–southwest orientation. Approximately two-thirds of the plot area is on the southeast slope, with the remainder on the northwest slope. Predominant tree species within the plot include evergreen broad-leaved species and subspecies like *Castanopsis carlesii*, *Castanopsis eyrei*, and *Schima superba* [9,35,36].

In accordance with the CTFS (Center for Tropical Forest Science) survey protocols, the entire plot was divided into 240 large quadrats (20 m × 20 m), and each large quadrat was further subdivided into 16 smaller plots (5 m × 5 m), totaling 3840 small plots. These smaller quadrats were used as work units to measure the relative position, DBH (diameter at breast height), and other individual attributes of all trees. From October to December 2013, during the first survey, we recorded species, relative position, DBH, height, and crown base height for all tree individuals with DBH ≥ 1 cm. Among these, one 5 m × 5 m or 1 m × 1 m subplot was selected in each large quadrat to survey shrubs, herbaceous plants, and lianas, recording their species, abundance, average height and cover (for shrubs and herbaceous plants), as well as basal diameter and length (for lianas). Specifically, for shrubs less than 1.3 m in height (the height at which DBH is measured) or with DBH < 1 cm, we only measured their average height and cover. The species listed in our study are exclusively woody plants. While we have also collected data on shrubs and herbaceous plants, these data were not included in this study. Our analysis only considers woody plants with DBH ≥ 1 cm.

The first census showed a total of 68,336 tree individuals (including branches and sprouts) with DBH ≥ 1 cm, belonging to 173 species, 88 genera, and 48 families. The co-dominant families included Fagaceae, Ericaceae, and Elaeocarpaceae, with co-dominant species including *Castanopsis carlesii*, *Castanopsis fordii*, *Castanopsis eyrei*, *Engelhardia roxburghiana*, *Syzygium buxifolium*, and *Schima superba*. No single species was overwhelmingly dominant (Table S1 in the Supplementary Files), and the stand structure indicated that the forest community in our study was still in the early stage of secondary succession because most tree individuals were saplings [9,36]. The second survey was conducted from September to December 2018. A total of 63,897 live trees (Table S2 in the Supplementary Files) were surveyed, including newly recruited individuals. Additionally, we noted that a total of 148 tree species (10.87% of the total number of tree) had died between 2013 and 2018 [9,36].

### 4.2. Relative Growth Rate

To evaluate tree productivity, we utilized the relative growth rate (RGR) of the tree’s wood volume. For each target tree, we calculated the wood volume (*V*) by employing a form factor of 0.5, which represents an average for young subtropical trees, where *V* = (π·d^2^/4)hf, d being the diameter at breast height (DBH), h the height of the tree, and f the form factor representing a cylinder [7,8]. The RGR of wood volume was determined using the following formula:

(1) $$RGR=\frac{log(V_{2}/V_{1})}{\left(t_{2}-{\;t}_{1}\right)}$$

where *V*_1_ and *V*_2_ represent the volumes of tree wood at the start *t*_2_ and end *t*_1_ of the study period from 2013 to 2018 (Figure S5 in the supporting information). We opted for RGR over the absolute growth rate due to the significant variation in the initial sizes of the trees under observation. RGR is a more reliable measure that is less influenced by the initial size differences among trees [7,37].

### 4.3. Neighborhood Diversity, Intraspecific/Interspecific Competition, and Test Scale

In our analysis, we developed a framework to elucidate the relationships between neighborhood diversity (NSR) and intraspecific (both conspecific neighborhood density, CND, and conspecific neighborhood competition index, CNCI) and interspecific competition represented by heterospecific neighborhood density, HND, and heterospecific neighborhood competition indices, HNCI, respectively, with the relative growth rate (RGR) of trees [7,38]. This methodology aimed to explore how these factors collectively influence tree growth [7,8,9,10,15,16].

Neighborhood species richness (NSR) was determined by counting the number of distinct tree species within a defined vicinity of each focal tree (Figure S6 in the Supplementary Files). Conspecific negative dependence (CND) and heterospecific negative dependence (HND) were assessed by examining the density of same-species and different-species trees surrounding a focal tree (Figure S7 in the Supplementary Files), respectively. The conspecific and heterospecific neighborhood competition indices (CNCI and HNCI) quantified the extent of resource competition, calculated by evaluating the DBH (diameter at breast height) area of neighboring trees of the same and different species (Figure S8 in the Supplementary Files), respectively. These indices, serving as a gauge for the abundance of competitors, were formulated as $NCI=\sum_{j\neq i}\frac{\pi D_{j}^{2}}{4}$, where *D_j_* represents the diameter at breast height (DBH) of neighboring trees [7]. CNCI includes only conspecific trees. For a given focal tree, CNCI is calculated as the sum of the DBH of all conspecific neighboring trees within a specified radius (from 5 m to 20 m). HNCI includes only heterospecific trees. Similarly, HNCI is calculated as the sum of the DBH areas of all heterospecific neighboring trees within a specified radius.

Neighborhood diversity and intra-/interspecific competition are highly spatial scale-dependent and closely related to the scale of the sampling radius [7,9,39]. In this study, the “neighborhood scale or local scale” (i.e., the test scale) was defined as the range with the focal tree species as the center and a radius less than 20 m to comprehensively evaluate the strength of the NSR effect at different spatial scales. In addition, at this neighborhood scale, our results could fully reflect the biological interaction relationship between species and avoid being confounded by the influence of habitat heterogeneity factors in the plot [17,33,40,41,42]. We calculated the NSR/CND/HND/CNCI/HNCI of focal tree species at different neighborhood scales in R-Studio (R 4.05, Boston, MA, USA)

### 4.4. Multiscale Neighborhood Effect Models for Tree Relative Growth Rate

To establish the relationship between neighborhood effects (NSR, CND, CNCI, HND, and HNCI) and relative growth rate (RGR), we employed linear mixed-effects models (LMMs). These models are tailored to elucidate the complex interactions influencing the annual growth rate of wood volume within a tree’s neighborhood. This approach offers a detailed understanding of how diversity, density dependence, and resource competition impact growth, enhancing our ability to predict growth dynamics across various species and environmental conditions.

In the linear mixed-effects model (LMM), *α* represents the intercept, indicating the baseline relative growth rate (RGR), and the *β* coefficients represent the fixed effects of the predictors (NSR, CND, CNCI, HND, and HNCI) on RGR. The random effects include species identity (random intercepts and slopes) and plot identity (random intercepts). Specifically, *c_s_* represents the random intercept for species s, accounting for the variability in the average growth rate across different species. *u*_1*s*_, *u*_2*s*_,*…*,*u*_5*s*_ are the species-specific random slopes for the predictors NSR, CND, CNCI, HND, and HNCI, respectively. These slopes capture the species-specific responses to the predictors, such as initial tree height, neighborhood competition, and neighborhood species richness, thereby accounting for inherent variability among species. *t_p_* represents the random effect of plot identity *p*, accounting for environmental heterogeneity among plots. These random effects account for intrinsic variability, thereby enhancing the robustness of the analysis. The error term (*ε*) is assumed to be normally distributed. The specific model structure is as follows:

(2) $$RGR_{i,s,p}=\alpha +(\beta_{1}+u_{1s})NSR_{i,s,p}+(\beta_{1}+u_{2s})CND_{i,s,p}+(\beta_{1}+u_{3s})CNCI_{i,s,p}+\left(\beta_{1}+u_{4s}\right)HND_{i,s,p}\;+(\beta_{1}+u_{5s})HNCI_{i,s,p}+DBH_{i,s,p}+c_{s}+t_{p}+\epsilon_{i,s,p}$$

We accounted for variation in abiotic growing conditions and species-specific effects by incorporating plot (quadrats), species identity, and neighborhood species effects (NSR, CND, CNCI, HND, and HNCI) into the random structure of our analysis. Specifically, a linear mixed-effects model (LMM) was employed, featuring both random intercepts and random slopes to account for variability among species and random intercepts alone to account for variability among plots [7,8,15,16]. The smallest plot scale of 5 × 5 m was deliberately chosen to effectively capture and control for tree dependencies. By including random intercepts for plots, we managed spatial dependencies and environmental heterogeneity within plots. Furthermore, by incorporating random slopes for species, we captured species-specific response variability, which further mitigated the issue of tree independence.

We utilized the “lme4 1.1-31” package for fitting LMMs [7,15,16]. According to the definitions of test scales in Section 2.3 of our study, we examined the effects of NSR, CND, CNCI, HND, and HNCI at distances of 2.5 m, 5 m, 10 m, 15 m, and 20 m from the focal tree species on RGR, thus establishing a multi-scale neighborhood effects model. The relative effect of each predictor (neighborhood effect) and their interactions is calculated as the ratio of its parameter estimate to the sum of all parameter estimates, expressed as a percentage [43]. Graphical and stand structural analyses were conducted using Excel and R-studio, utilizing R version 4.05 with packages, ‘vegan’ 2.5-7, and ‘ads’ 1.5-5.

## 5. Conclusions

The elucidation of the relationship between species diversity and ecosystem productivity has remained a cornerstone in ecological research, with significant implications for biodiversity conservation and ecosystem management strategies [1,44,45]. Our study observed a significant positive correlation between neighborhood-scale species diversity and the relative growth rate (RGR) of individual trees within a secondary forest ecosystem. Confirming our initial hypothesis (H1), our findings underscore a significant positive correlation between neighborhood diversity and individual tree growth, reaffirming the importance of biodiversity in fostering ecosystem productivity. Notably, we observed a scale-dependent effect, wherein the biodiversity–productivity relationship exhibited greater variability at smaller scales but stabilized into a consistent positive correlation as spatial scale increased. Our investigation into intraspecific and interspecific competition further elucidates the mechanisms driving the observed patterns. At smaller scales, intraspecific competition, as indicated by the conspecific neighbor density (CND) and conspecific neighbor competition index (CNCI), exerted a notable negative impact on relative growth rate (RGR), reflecting the dominance of conspecific density-dependent effects. In contrast, interspecific competition, represented by the heterospecific neighbor density (HND) and heterospecific neighbor competition index (HNCI), exhibited insignificance or a lesser influence on RGR, suggesting a lower intensity compared to intraspecific competition. Combining these insights, it becomes evident that intraspecific competition plays a pivotal role in shaping individual tree growth, especially at smaller scales, while interspecific competition contributes less significantly. However, as spatial scale increases, the relative importance of neighborhood species richness (NSR) becomes more pronounced, contributing to a positive diversity–productivity relationship. This shift highlights the complex interplay of local density-dependent interactions and spatial scale in modulating ecosystem functioning.

It is important to note that diameter at breast height (DBH) is a widely used and accepted method for quantifying competition, especially due to its robust and reliable nature in large-scale surveys [7,8,9,10,15,16,17]. Moreover, DBH is one of the most precise measurable variables obtainable through traditional, non-automated field surveys. Other variables, like tree height and crown width, often encounter significant error due to factors like stand density and individual tree occlusion, making them less reliable, especially in expansive surveys. However, we acknowledge the potential limitations of using only DBH to evaluate resource competition in diverse ecosystems, particularly in multispecies evergreen forest communities. Future forest surveys should consider employing more precise instruments, such as LiDAR (Light Detection and Ranging), to obtain detailed forest structure data. Such advanced technologies can offer more precise measurements of tree height, canopy volume, and spatial distribution, thereby improving our ability to quantify competition and enhancing our understanding of biodiversity and ecosystem productivity relationships.

Overall, our study contributes to a deeper understanding of how biodiversity influences ecosystem productivity across different scales, emphasizing the need for a nuanced approach in ecosystem management and conservation efforts. By unraveling the intricate mechanisms underlying the biodiversity–productivity relationship, our findings offer insights for fostering sustainable stewardship of natural resources and guiding forest restoration initiatives in degraded ecosystems.

## Data Availability

The forest census data are available from the corresponding author on reasonable request.

## Supplementary Materials

The following supporting information can be downloaded at https://www.mdpi.com/article/10.3390/plants13141994/s1, Figure S1. Relationship between species richness and relative growth rate (RGR) among all species. It displays annual diversity–RGR relationships for each of the 158 observed species at 20 m. Different species are represented by different colors in the lines. Predicted RGR are back-transformed from the linear mixed model as described in the text, and all diversity effects were Z-score transformed at quantification. Figure S2. Multiscale relationship between neighborhood effect (NSR, CND, CNCI, HND, and HNCI) and relative growth rate (RGR) among all species. The annual neighborhood effect–RGR relationship across the 158 observed species is represented by lines of different colors (refer to Figure S1), with solid lines indicating a positive relationship and dashed lines indicating a negative one. The predicted values of RGR are obtained by back-transforming from the linear mixed models, with all diversity effects quantified by Z-score transformation. In this Figure, it is noted that Figure 3, Figure 5, and Figure 7 in the main text only present the prediction results for positive Z-scores, whereas the original results are displayed here. Figure S3. Schematic representation of the location of the study area. The red dots in the figure represent the location of the subtropical evergreen broad-leaved forest plot in the Wuyi Mountains, China. Figure S4. The spatial pattern of all tree individuals in the evergreen broad-leaved secondary forest dynamic observation site. Circles represent the locations of individual trees, whose sizes are proportional to the tree DBH (diameter at breast height); dark green circles represent tree individuals in first census in 2013, and steel blue circles represent tree individuals in 2018. Figure S5. The spatial pattern of relative growth rate (RGR) in the evergreen broad-leaved secondary forest dynamic observation site. This figure represents the annual relative growth rate (RGR) of tree individuals during 2013–2018. RGR decreases gradually as the color transitions from red to blue. The spatial intensity was estimated using an Epanechnikov kernel with a bandwidth of 10 m. Figure S6. The spatial pattern of neighborhood diversity (NSR). This figure represents the neighborhood species richness (NSR) of tree individuals in 2018. NSR decreases gradually as the color transitions from red to blue. The spatial intensity was estimated using an Epanechnikov kernel with a bandwidth of 10 m. Figure S7. The spatial intensities of neighborhood density. This figure represents the intensity of conspecific and heterospecific neighborhood densities of tree individuals in 2018. The intensity decreases gradually as the color transitions from red to blue. The spatial intensity was estimated using an Epanechnikov kernel with a bandwidth of 10 m. Figure S8. The spatial pattern of neighborhood size (DBH). This figure represents the intensity of neighborhood size (DBH) of tree individuals in 2018. DBH decreases gradually as the color transitions from red to blue. The spatial intensity was estimated using an Epanechnikov kernel with a bandwidth of 10 m. Table S1. Basic characteristics of the 28 co-dominant tree species in the subtropical evergreen broad-leaved forest plot in the Wuyi Mountains, China. Table S2. Forest dynamic in the subtropical evergreen broad-leaved forest plot in the Wuyi Mountains, China for the years 2013 and 2018.
