# Supplementary material for: Neighborhood Diversity Promotes Tree Growth in a Secondary Forest: The Interplay of Intraspecific Competition, Interspecific Competition, and Spatial Scale
Source: Plants (Basel). 2024 Jul 21;13(14):1994. doi: 10.3390/plants13141994 (PMC11280550; doi:10.3390/plants13141994)
Supplement: Supplementary file 1 [file plants-13-01994-s001.zip › plants-3055479-supplementary.pdf]

Supplementary Files

Figure S1. Relationship Between Species Richness and Relative Growth Rate (RGR) among all species. It displays annual diversity–RGR relationships for each of the 158 observed species at 20 m. Different species are represented by different colors in the lines. Predicted RGR are back-transformed from the linear mixed model as described in the text, and all diversity effects were Z-score transformed at quantification.

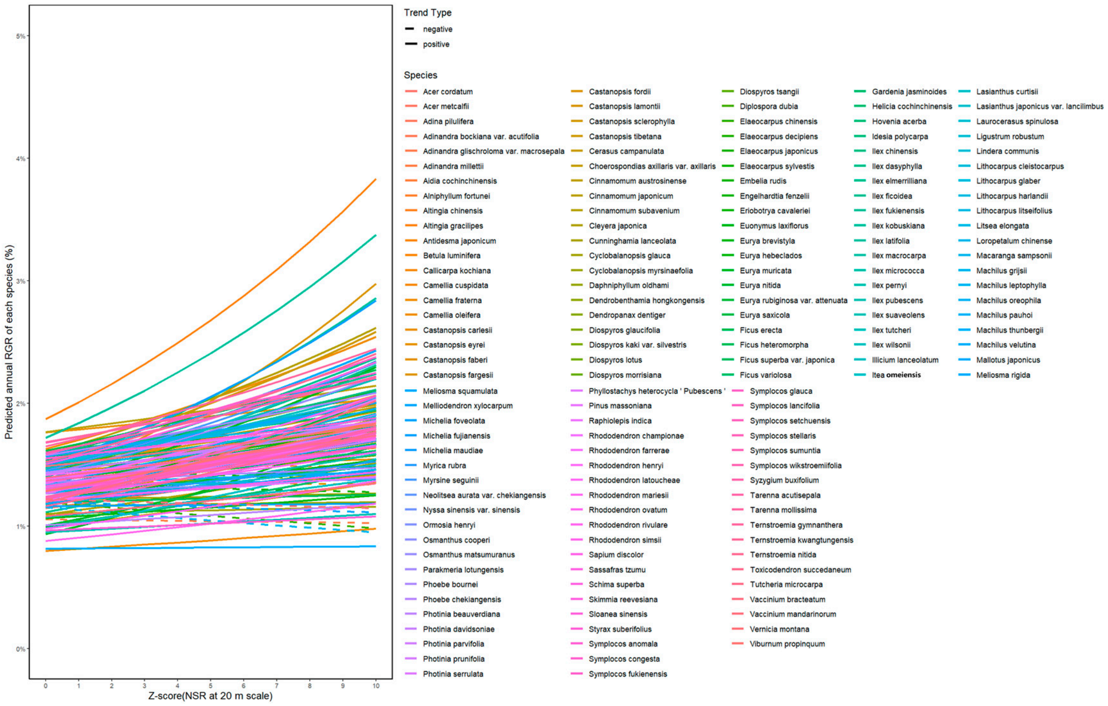

Figure S2. Multiscale Relationship Between neighborhood effect (NSR, CND, CNCI, HND, HNCI) and Relative Growth Rate (RGR) Among All Species. The annual neighborhood effect - RGR relationship across the 158 observed species is represented by lines of different colors (refer to Figure S1), with solid lines indicating a positive relationship and dashed lines indicating a negative one. The predicted values of RGR are obtained by back-transforming from the linear mixed models, with all diversity effects quantified by Z-score transformation. In this Figure, it is noted that Figures 3, 5, 7 in the main text only present the prediction results for positive Z-scores, whereas the original results are displayed here.

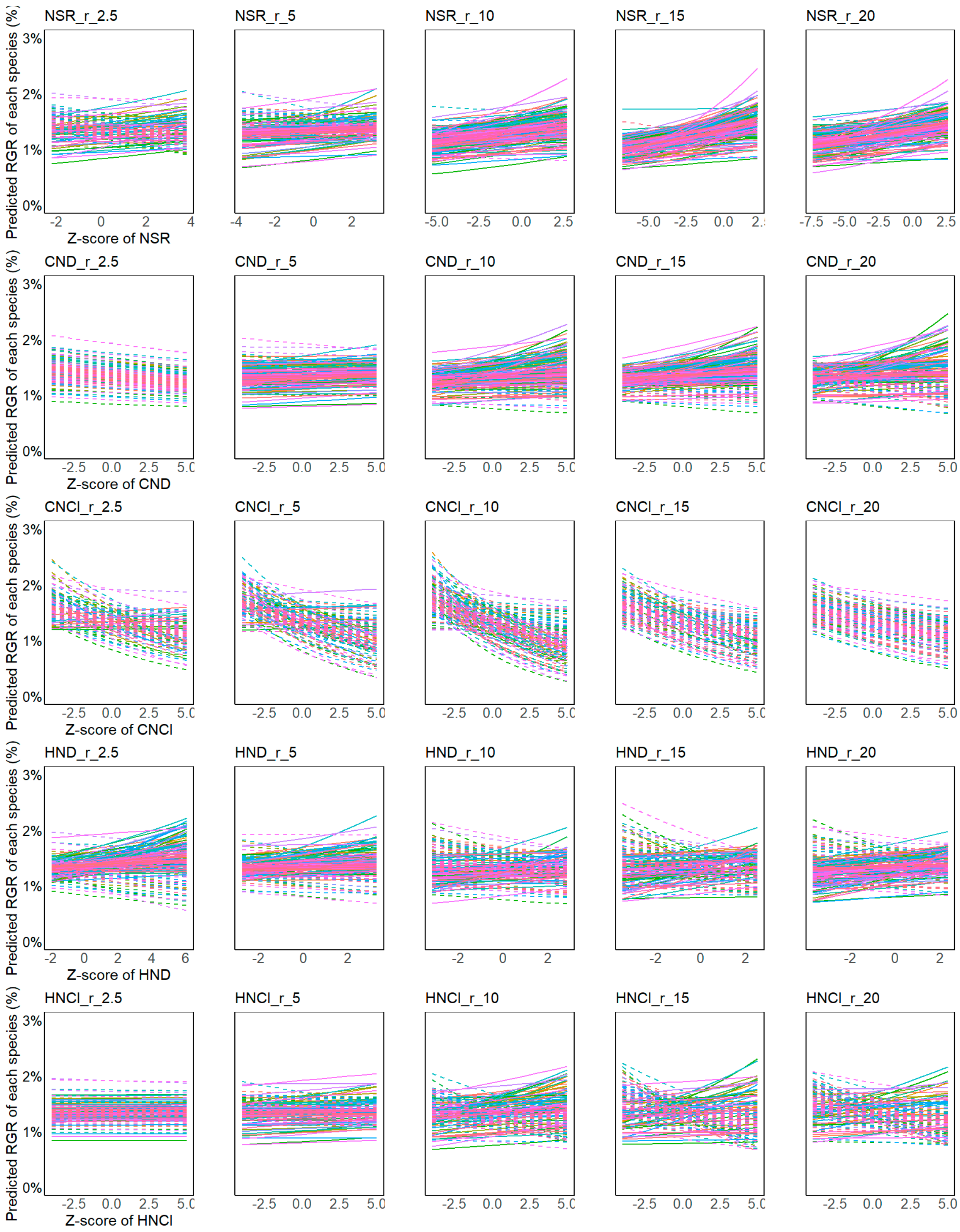

Figure S3. Schematic representation of the location of the study area. The red dots in the figure represent the location of Subtropical Evergreen Broad-Leaved Forest plot in the Wuyi Mountains, China.

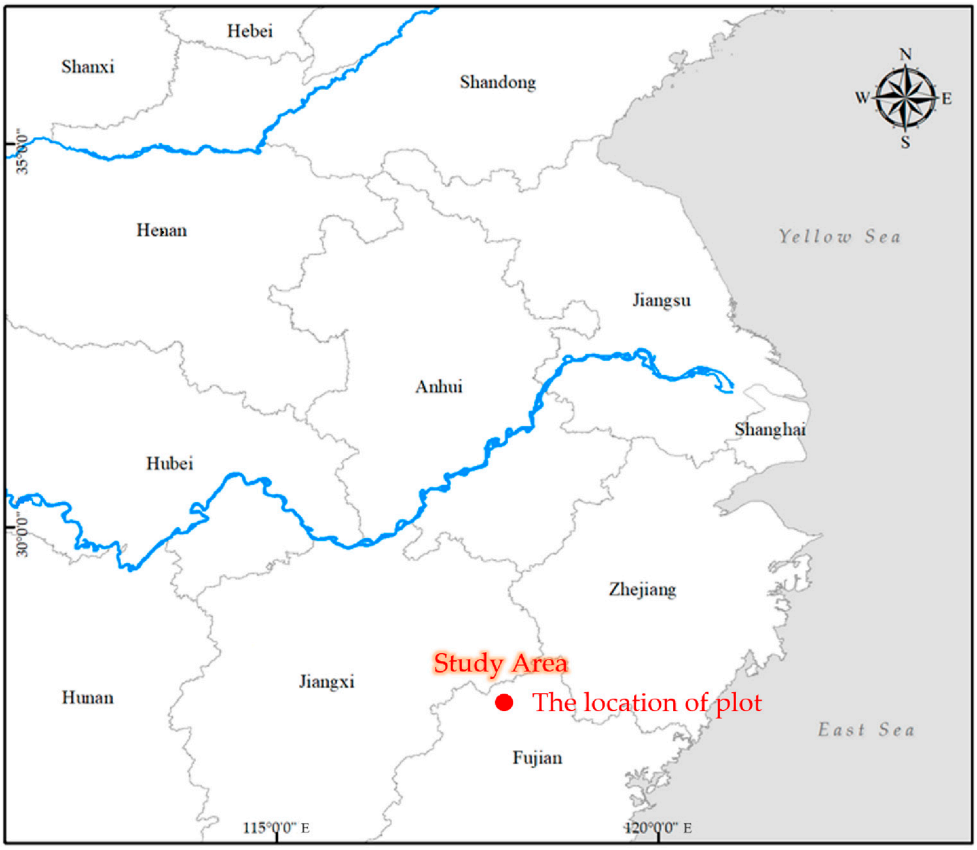

Figure S4. The spatial pattern of all tree individuals in evergreen broad-leaved secondary forest dynamic observation site. Circles represent the locations of individual trees, whose sizes are proportional to the tree DBH (diameter at breast height); dark green circles represent tree individuals in first census in 2013 censor, and steel blue circles represent tree individuals in 2018.

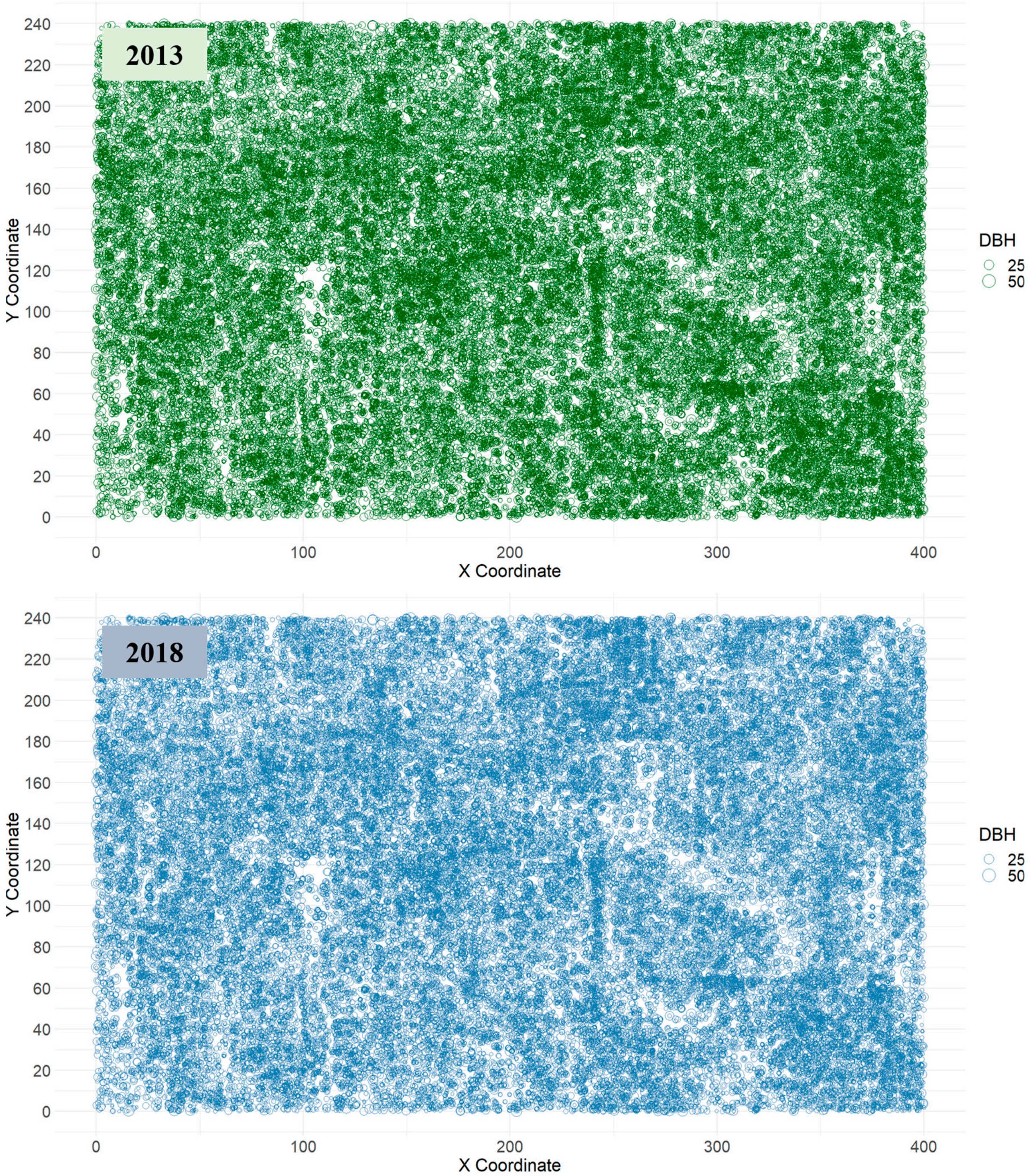

Figure S5. The spatial pattern of relative growth rate (RGR) in evergreen broad-leaved secondary forest dynamic observation site. This figure represents the annual relative growth rate (RGR) of tree individuals during 2013–2018. RGR decreases gradually as the color transitions from red to blue. The spatial intensity was estimated using an Epanechnikov kernel with a bandwidth of 10 m (Baddeley et al., 2015; Zhang et al., 2021a).

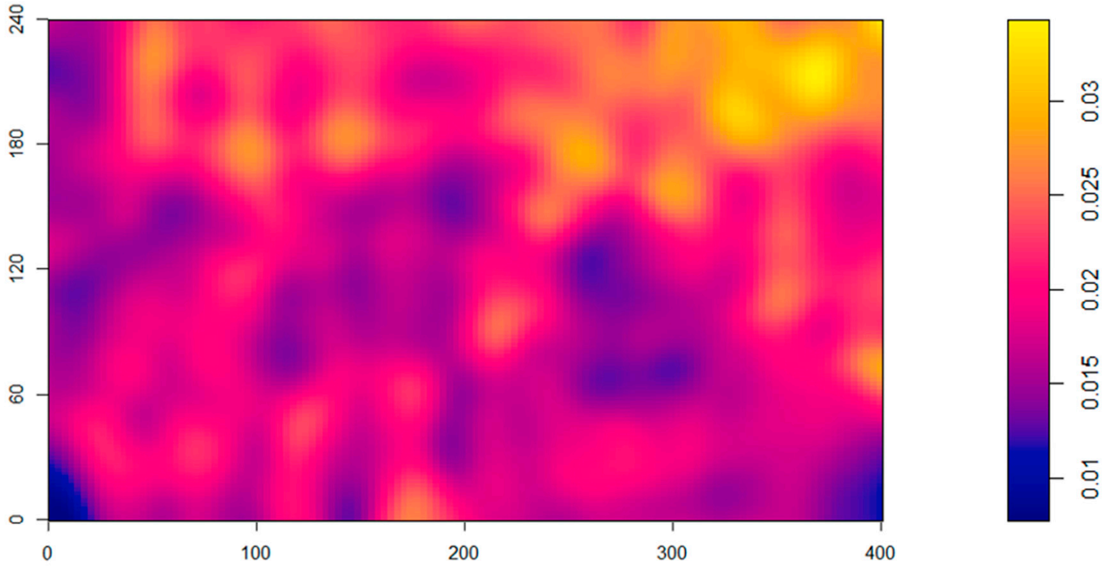

Figure S6. The spatial pattern of neighborhood diversity (NSR). This figure represents the neighborhood species richness (NSR) of tree individuals in 2018. NSR decreases gradually as the color transitions from red to blue. The spatial intensity was estimated using an Epanechnikov kernel with a bandwidth of 10 m (Baddeley et al., 2015; Zhang et al., 2021a).

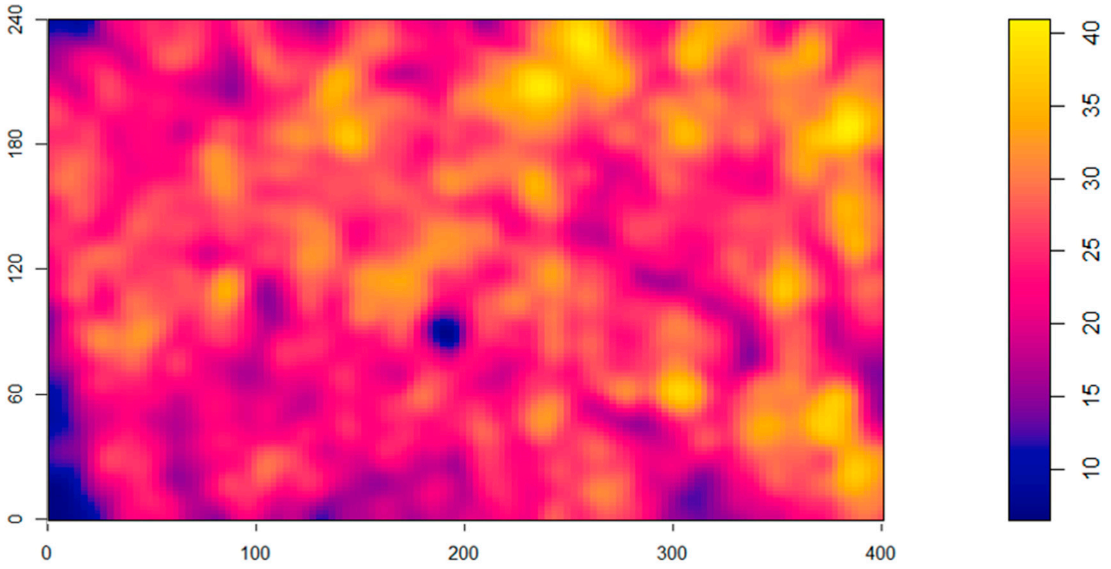

Figure S7. The spatial intensities of neighborhood density. This figure represents the intensity of conspecific and heterospecific neighborhood density of tree individuals in 2018. The intensity decreases gradually as the color transitions from red to blue. The spatial intensity was estimated using an Epanechnikov kernel with a bandwidth of 10 m (Baddeley et al., 2015; Zhang et al., 2021a).

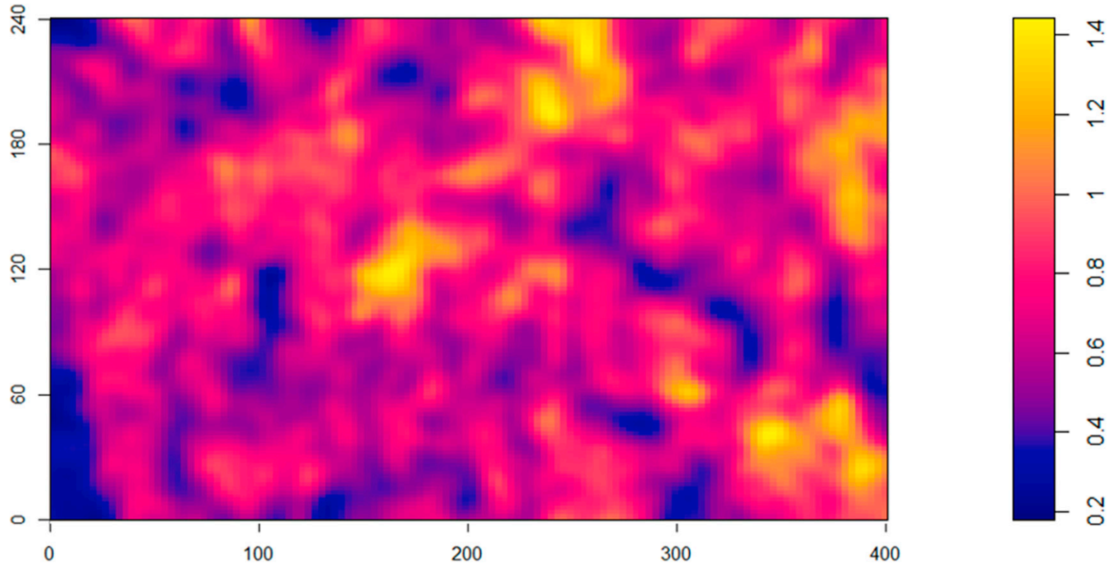

Figure S8. The spatial pattern of neighborhood size (DBH). This figure represents the intensity of neighborhood size (DBH) of tree individuals in 2018. DBH decreases gradually as the color transitions from red to blue. The spatial intensity was estimated using an Epanechnikov kernel with a bandwidth of 10 m (Baddeley et al., 2015; Zhang et al., 2021a).

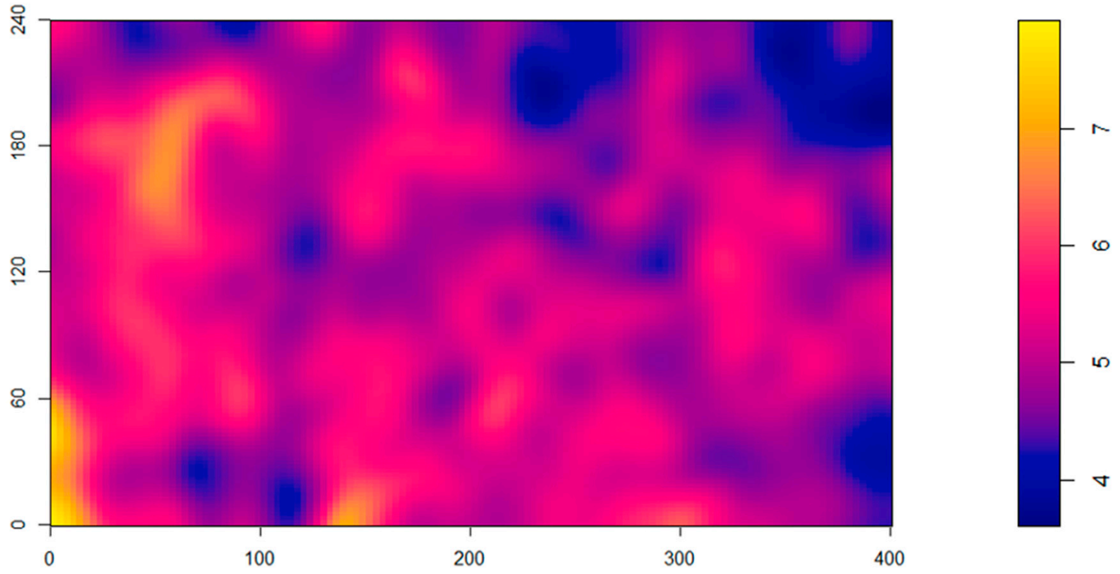

Table S1. Basic characteristics of the 28 co-dominant tree species in the Subtropical Evergreen Broad-Leaved Forest plot in the Wuyi Mountains, China

Species	Importance Value (%)	Average of DBH (cm)	Average of Height (m)
Castanopsis carlesii	7.16	11.65 ± 0.26	8.44 ± 0.12
Castanopsis fordii	5.29	7.52 ± 0.13	7.04 ± 0.08
Castanopsis eyrei	4.57	11.44 ± 0.27	8.60 ± 0.12
Engelhardia roxburghiana	4.37	7.57 ± 0.14	7.15 ± 0.08
Syzygium buxifolium	3.67	2.89 ± 0.05	4.50 ± 0.03
Schima superba	3.16	7.96 ± 0.20	7.76 ± 0.12
Rhododendron henryi	2.91	3.52 ± 0.05	4.85 ± 0.04
Eurya muricata	2.78	2.53 ± 0.04	4.02 ± 0.03
Altingia gracilipes	2.66	6.17 ± 0.18	6.54 ± 0.11
Itea omeiensis	2.89	2.83 ± 0.03	4.89 ± 0.04
Michelia fujianensis	2.49	3.72 ± 0.06	4.60 ± 0.05
Castanopsis faberi	2.79	13.34 ± 0.37	9.55 ± 0.17
Elaeocarpus japonicus	2.44	13.03 ± 0.26	11.09 ± 0.15
Camellia cuspidata	2.33	2.06 ± 0.02	3.68 ± 0.04
Lithocarpus harlandii	2.00	7.19 ± 0.18	7.18 ± 0.11
Castanopsis fargesii	2.25	11.57 ± 0.40	9.06 ± 0.20
Elaeocarpus chinensis	2.12	8.58 ± 0.20	8.76 ± 0.13
Ormosia henryi	1.75	3.82 ± 0.09	5.55 ± 0.08
Alniphyllum fortunei	1.64	13.44 ± 0.28	12.32 ± 0.16
Eurya hebeclados	1.55	2.49 ± 0.03	3.82 ± 0.04
Symplocos congesta	1.43	3.56 ± 0.09	4.94 ± 0.07
Machilus grijsii	1.65	2.02 ± 0.04	3.92 ± 0.08
Michelia maudiae	1.35	4.34 ± 0.14	5.64 ± 0.11
Cyclobalanopsis myrsinifolia	1.36	6.84 ± 0.37	6.74 ± 0.16
Dendropanax dentiger	1.31	6.33 ± 0.17	6.75 ± 0.11
Cinnamomum japonicum	1.20	3.52 ± 0.12	5.04 ± 0.09
Rhododendron ovatum	1.20	3.84 ± 0.07	5.15 ± 0.06
Ilex pubescens	1.01	1.94 ± 0.04	3.64 ± 0.06

Table S2. Forest Dynamic in the Subtropical Evergreen Broad-Leaved Forest plot in the Wuyi Mountains, China for the Years 2013 and 2018

Year	Number Of live trees	Species richness	Recruit Rate (%)	Mortality rate (%)
2013	68336	173	4.37	10.87
2018	63897	174		

Note: The highest number of species shown in the figures of our study is 158 because we divided the entire plot (9.6 ha) into several large quadrats (20 m × 20 m), which were further subdivided into 16 smaller quadrats (5 m × 5 m) each, and focused on tree-to-tree neighborhood scales in our research. Thus, the neighborhood test scale was up to large quadrats (20 m × 20 m), and at most, only 158 species could be shown. That is, the maximum number of species detectable within the 240 20 m × 20 m quadrats is 158. Some species were not included in the detection results due to low numbers and dispersed distribution, but this does not affect the overall analysis. For example, adjusting the test range to 50 m × 50 m would detect all 173 species, supporting the classic species-area relationship theory.
